# The incidence and risk factors of atrial high‐rate episodes in patients with a dual‐chamber pacemaker

**DOI:** 10.1002/joa3.13143

**Published:** 2024-09-03

**Authors:** Son Khac Le Nguyen, Dung Ngoc Kieu, Phuong Le Uyen Tran, Chuong Khac Thien Nguyen, Toan Quang Dang, Chieu Van Ly, Sy Van Hoang, Thuc Tri Nguyen

**Affiliations:** ^1^ Department of Arrhythmia Treatment Cho Ray Hospital Ho Chi Minh City Vietnam; ^2^ Department of Cardiology Cho Ray Hospital Ho Chi Minh City Vietnam; ^3^ Department of Internal Medicine University of Medicine and Pharmacy at Ho Chi Minh City Ho Chi Minh City Vietnam

**Keywords:** atrial high‐rate episodes (AHRE), global longitudinal strain (GLS), subclinical atrial fibrillation

## Abstract

**Background and Objectives:**

Cardiovascular implantable electronic devices can detect atrial high‐rate episodes (AHREs). However, the predictors of clinically relevant AHREs have not been well identified.

**Methods:**

This prospective study included 145 patients (median age 64.5 ± 16.4 years, 53.1% females) without atrial fibrillation (AF) from December 2020 to January 2022. AHREs were defined as a programmed atrial detection rate >190 beats per minute. Cox regression analysis was used to identify the risk factors of AHREs.

**Results:**

During 6 months of follow‐up, AHREs occurred in 30.3% of patients. Multivariable Cox regression analysis showed factors related to development of AHREs including using anti‐arrhythmic drugs (AAD) before implantation (Hazard ratio (HR) 7.71; 95% confidence interval [95% CI], 2.58–23.02, *p* < .001), history of paroxysmal supraventricular tachycardia (PSVT; HR 2.45; [95% CI], 1.18–5.09, *p* = .016), the percentage of premature atrial contraction (PAC) on 24‐h Holter electrocardiogram (ECG) monitoring (HR 1.008; [95% CI], 1.003–1.014, *p* = .003), and left ventricular global longitudinal strain (GLS‐LV; HR 0.92;[95% CI], 0.84–0.99, *p* = .049).

**Conclusions:**

This study showed that a history of PSVT and using AAD, the percentage of PAC on 24‐h Holter ECG monitoring, and GLS‐LV were the independent predictors of new‐onset AHREs.

## Graphical abstract

This prospective study identified a 30.3% incidence of atrial high‐rate episodes (AHREs) in patients with dual‐chamber pacemakers. History of anti‐arrhythmic drug use, PSVT, premature atrial contractions, and left ventricular global longitudinal strain were independent predictors of AHREs.

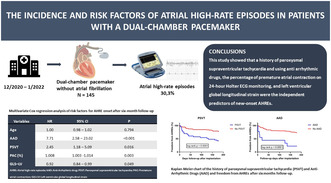

## 1 INTRODUCTION

Atrial fibrillation is the most common chronic supraventricular arrhythmia in clinical practice.^1^ We already know that atrial fibrillation increases stroke risk and cardiovascular death.^2^, ^3^, ^4^ Clinical atrial fibrillation usually precedes subclinical atrial tachyarrhythmias or atrial high‐rate episodes.^5^ Where atrial high‐rate episodes (AHRE) last longer than 5 min, it is found to increase the risk of stroke significantly. It must be emphasized that patients with AHRE should be evaluated for clinical atrial fibrillation and an increased risk of stroke.^6^ While traditional methods such as electrocardiogram (ECG) and Holter ECG may not be effective for detecting these silent atrial tachycardias, new techniques such as wearable ECG devices or smartphone watches demonstrated a great ability to detect them.^7^ In addition, cardiac implantable electrocardiographic devices such as pacemakers, implantable cardioverter‐defibrillators, cardiac resynchronization therapy devices, and implantable long‐term ECG monitors can detect episodes of atrial or ventricular arrhythmias early even when the patient is asymptomatic.^8^ Patients with implantable ECG devices often have comorbidity diseases that predispose them to atrial fibrillation. These devices then continuously monitor the patient's ECG and detect silent AHRE. AHRE detected by these devices in patients without a prior history of atrial fibrillation can predict long‐term fatal outcomes and have been shown to increase the risk of clinical atrial fibrillation, increased stroke risk, and systemic thrombosis.^6^, ^9^, ^10^ Many risk factors, such as age, hypertension, diabetes, heart failure, vascular disease, heart valve disease, increase the risk of atrial fibrillation.^11^ However, whether these factors are independent predictors for AHRE is unknown.^12^, ^13^ Therefore, this study was designed to evaluate the incidence and risk factors associated with the occurrence of AHRE in patients with dual‐chamber pacemakers at Cho Ray Hospital.

## 2 METHODS

### 2.1 Study design

**FIGURE 1:**
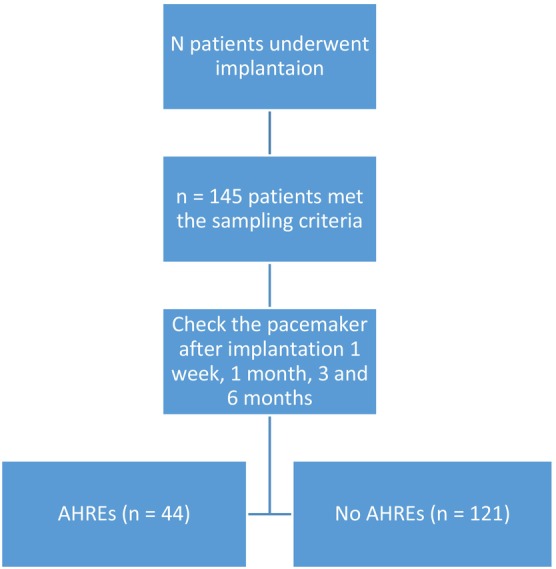
Flow diagram showing study participants.

“The Incidence and Risk Factors of Atrial High‐Rate Episodes in Patients with A Dual‐Chamber Pacemaker” is a single‐center, prospective cohort study conducted at the Department of Arrhythmia Treatment—Cho Ray Hospital. The study started enrolling patients in December 2020 and ended in January 2022 (Figure 1). All patients who met the inclusion criteria were continuously sampled, and there were no exclusion criteria for dual‐chamber pacemaker implantation at the hospital.

### 2.2 The inclusion and exclusion criteria

The study population included all patients with an indication for permanent dual‐chamber pacemaker implantation according to the 2018 ACC/AHA/HRS Guideline on the Evaluation and Management of Patients with Bradycardia and Cardiac Conduction Delay: A Report of the American College of Cardiology/American Heart Association Task Force on Clinical Practice Guidelines and the Heart Rhythm Society. A total of 145 consecutive patients were enrolled and implanted with a St. Jude Medical (Assurity PM2240), Medtronic (Ensura DR MRI SureScan Pacing System, Medtronic, Inc), Biotronik (Evity 8 DR‐T; Biotronik, Berlin, Germany) dual‐chamber rate‐adaptive pacemaker for sick sinus syndrome (sinus bradycardia, sinus pause ≥3 s, tachycardia‐bradycardia syndrome, sinus node dysfunction, and chronotropic incompetence) or atrioventricular block (high degree/complete atrioventricular block) or vasovagal. We collected data on the basic demographic characteristics, clinical information, 12‐lead ECG, blood tests, echocardiographic parameters such as left ventricular systolic and diastolic function, and pre‐implantation medications. Left ventricular global longitudinal strain (GLS‐LV) was measured by speckle tracking echocardiography using a Vivid E95 vendor (General Electric, Fairfield, CT, USA). Holter ECG, stress ECG, and transthoracic echocardiography were performed before pacemaker implantation. The exclusion criteria were documented clinical atrial fibrillation recorded on standard 12‐lead ECG, Holter ECG, or stress ECG. We also excluded from the sample patients who died within 6 months of implantation or those patients who lost tracking and cannot collect data during that time. Patients were interrogated with the programmer at the Arrhythmia Clinic at 1 week, 1, 3, and 6 months after implantation. We collected atrial and ventricular pacing rates, atrial and ventricular pacing parameters, threshold, sensing and impedance parameters. According to “Device‐detected subclinical atrial tachyarrhythmias: definition, implications and management—an European Heart Rhythm Association (EHRA) consensus document, endorsed by Heart Rhythm Society (HRS), Asia Pacific Heart Rhythm Society (APHRS) and Sociedad Latinoamericana de Estimulacion Cardıaca y Electrofisiologıa (SOLEACE) 2017”^14^ AHRE are defined as atrial tachyarrhythmia episodes with rate >190 beats/min detected by cardiac implantable electronic devices and lasting at least 10 beats. We also collected the duration of AHRE episodes and the date that AHRE onset. We defined a history of paroxysmal supraventricular tachycardia (PSVT) as any atrial tachycardia (including multifocal atrial rhythm) with either a rate less than 190 beats/min or a short episode lasting less than 10 beats recorded on 24‐h Holter ECG monitoring before implantation.

### 2.3 Statistical analysis

Continuous variables were presented as mean ± standard deviations for normally distributed values or medians and interquartile interval (IQI) or mean and minimum—maximum value for non‐normally distributed values and categorical variables as numbers and percentages in each group. The baseline characteristics of the two groups were compared using Student's *t*‐test or Mann–Whitney *U*‐tests for continuous variables and Pearson's Chi‐squared or Fisher's exact tests for categorical variables. We used a Cox regression model for multivariate analysis to assess predictors of AHREs after adjusting for other clinical events. Based on age, gender, and body mass index (BMI), we built a multivariate analysis model by adding variables that were *p* values <.05 into the univariate models. Statistical analyses were performed using SPSS software ver. 22.0 (IBM, Armonk, NY, USA). Two‐tailed *p* values <.05 indicated statistical significance.

### 2.4 Ethical issues in research

This study has been approved by the ethics committee on biomedical research at Pham Ngoc Thach University of Medicine according to Official Letter No. 445/HDĐ‐ĐHYKPNT dated December 11, 2020. We conducted the study to serve patients and society, not for self‐interest, not to harm the patient's health and spirit. The study was conducted with respect for the patient's privacy and with the patient's consent. All patient information is processed as data, without identifying the patient. The laboratory tests in the study were routine, non‐invasive tests. Our study does not interfere with the treatment process or affect the treatment results and the patient's psychology.

## 3 RESULTS

### 3.1 Baseline demographic characteristics

From December 1, 2020 to January 31, 2022, we recruited 145 patients and followed up to 6 months after implantation (Table 1). The average age was 64.5 ± 16.4; females accounted for 53.1%. The indications due to sick sinus syndrome accounted for 58.6%, complete heart block accounted for 37.9%, and the rest was vasovagal syncope. The average CHA2DS2_VASc scores were 2.00 [1.00–3.00]. Among the patients participating in the study, we recorded co‐morbidities; the highest rate was hypertension (57.2%) and ischemia heart disease (44.4%) with 1/5 of the population having structural heart disease (20%) and nearly 1/5 of the population having diabetes (18.6%). There were no significant differences between the two groups.

**TABLE 1:** Baseline characteristics of the overall population (*n* = 145).

Characteristics	Total (*N* = 145)	AHRE (*N* = 44)	No AHRE (*N* = 101)	*p*
Female *n* (%)	77 (53.10%)	26 (55.09%)	51 (50.50%)	.340
Age (years)	64.5 ± 16.4	64.9 ± 17.1	64.2 ± 16.2	.809
BMI (kg/m^2^)	22.7 ± 3.2	22.4 ± 2.7	22.9 ± 3.7	.400
CHA2DS2_Vasc	2.0 [1.0–3.0]	2.0 [1.0–3.8]	2.0 [2.0–3.0]	.868
Heart failure *n* (%)	10 (6.9%)	1 (2.3%)	9 (8.9%)	.155
CAD *n* (%)	64 (44.4%)	19 (43.2%)	45 (44.6%)	.968
Hypertension *n* (%)	83 (57.2%)	27 (61.4%)	56 (55.5%)	.508
Diabetes *n* (%)	27 (18.6%)	5 (11.4%)	22 (21.8%)	.138
Stroke/TIA *n* (%)	11 (7.6%)	2 (4.6%)	9 (8.9%)	.361
PAD *n* (%)	3 (2.1%)	0 (0.0%)	3 (2.9%)	.248
AVB *n* (%)	55 (37.9%)	12 (27.3%)	43 (42.6%)	.213
SSS *n* (%)	85 (58.6%)	30 (68.2%)	55 (54.5%)	.213
Ap (%)	15.0 [0.0–69.7]	7.9 [0.5–63.4]	22.3 [0.0–75.0]	.567
Vp (%)	25.0 [0.0–100.0]	13.5 [0.0–99.3]	45.0 [0.0–100.0]	.702
ACEI/ARB *n* (%)	74 (51.4%)	23 (52.3%)	51 (50.5%)	.888
MRA *n* (%)	11 (7.6%)	1 (2.3%)	10 (9.9%)	.108
Statin *n* (%)	81 (56.3%)	27 (61.4%)	54 (53.5%)	.412
AAD *n* (%)	9 (6.2%)	7 (15.9%)	2 (1.9%)	.001
PSVT *n* (%)	33 (22.8%)	19 (43.2%)	14 (13.9%)	<.001
PAC (%)	0.9 [0.0–31.0]	2.5 [0.0–31.0]	0.3 [0.0–7.4]	<.001
eGFR (mL/min/1.73 m^2^)	78.9 ± 23.9	78.3 ± 23.1	79.1 ± 24.3	.842
CRP (mg/L)	3.3 [0.4–12.5]	2.5 [0.2–15.9]	3.5 [0.4–10.5]	.972
HGB (g/L)	127.7 ± 16.2	128.4 ± 14.3	127.4 ± 17.0	.725
WBC (G/L)	8.4 ± 2.9	8.5 ± 3.0	8.4 ± 2.8	.764
LAd (mm)	34.9 ± 6.9	33.4 ± 7.2	35.7 ± 6.6	.069
LAVI (mL/m^2^)	21.1 ± 11.6	19.7 ± 8.5	21.7 ± 12.7	.369
E (cm/s)	74.5 ± 30.3	76.4 ± 31.2	73.6 ± 29.9	.615
E/A	1.2 ± 0.8	1.2 ± 0.8	1.2 ± 0.9	.798
E/E'Ave	8.9 ± 4.8	8.2 ± 4.0	9.3 ± 5.1	.256
DT (ms)	209.0 ± 85.4	198.0 ± 67.4	213.9 ± 92.2	.342
LVEDD (mm)	48.1 ± 6.3	48.1 ± 5.8	48.1 ± 6.6	.965
LVESD (mm)	31.4 ± 6.0	31.0 ± 5.1	31.5 ± 6.4	.693
LVEF (%)	63.5 ± 10.1	65.2 ± 7.7	62.8 ± 11.0	.195
GLS‐LV (%)	−18.6 ± 4.6	−19.9 ± 4.1	−18.0 ± 4.6	.036
GLS‐LV ≤ −18% *n* (%)	70 (59.3%)	24 (54.6%)	46 (45.5%)	.065

Abbreviations: AAD, anti‐arrhythmic drug; ACEI/ARB, angiotensin‐converting enzyme inhibitor/angiotensin receptor blocker; AHRE, atrial high‐rate episode; Ap, atrial pacing; AVB, atrial ventricular block; BMI, body mass index; CAD, coronary artery disease; CRP, C‐reactive protein; DT, declaration time; eGFR, estimated glomerular filtration rate; GLS‐LV, left ventricular global longitudinal strain; HGB, hemoglobin; LAd, left atrial diameter; LAVI, left atrial volume index; LVEDD, left ventricular end‐diastolic dimension; LVEF, left ventricular ejection fraction; LVESD, left ventricular end‐systolic dimension; MRA, mineralocorticoid receptor antagonists; PAC, premature atrial contraction; PAD, peripheral artery disease; PSVT, paroxysmal supraventricular tachycardia; SSS, sick sinus syndrome; TIAs, transient ischemic attack; Vp, ventricular pacing; WBC, white blood cell.

Regarding pre‐implantation medication, there was a statistically significant difference. AHRE was higher in the group of AAD using with *p* = .005. There were nine patients using AAD, including three patients with ventricular premature contraction using amiodarone and six patients diagnosed with tachycardia‐bradycardia syndrome using flecainide.

Regarding pre‐implantation arrhythmias, there was a significant difference between the two groups: the history of the PSVT group had a higher rate of AHRE (43.2% vs. 13.9%) with *p* < .001. Regarding the percentage of PAC on Holter ECG before implantation, there was a higher rate of AHRE in the group that had PAC (2.53% vs. 0.33%) with *p* < .001.

Our study collected data about echocardiographic parameters. The GLS‐LV was higher in the AHRE group compared with the No‐AHRE group (−19.9 ± 4.1 in the AHRE group vs. −18.0 ± 4.6 in the No‐AHRE group, *p* = .036). However, when subgroup analysis was divided into two groups, GLS_LV ≤−18% and >−18%, there was no statistically significant difference between the groups, *p* = .065.

### 3.2 Characteristics of AHRE (Table 2)

**TABLE 2:** Characteristics of AHRE.

Characteristics	Results
AHRE *n* (%)	44 (30.3%)
AHRE >6 min *n* (%)	18 (40.9%)
AHRE rate (bpm)	235.0 ± 91.3
AHRE duration (s) [25%—75%]	76.5 [16.0–762.0]
Day first‐time onset AHRE	28.5 ± 14.5
AHRE duration	
<5.5 h *n* (%)	36 (81.8%)
5.5–24 h *n* (%)	4 (9.1%)
≥24 h *n* (%)	4 (9.1%)

Abbreviation: AHRE, atrial high‐rate episode.

The incidence of AHRE is 30.3% (44/145). AHRE lasting more than 6 min accounted for 40.9%. The average rate of AHRE was 235.0 ± 91.3 bpm. The average day first‐time onset of AHRE was 28.5 ± 14.5.

### 3.3 Univariate cox regression analysis of the risk factors of AHRE

**FIGURE 2:**
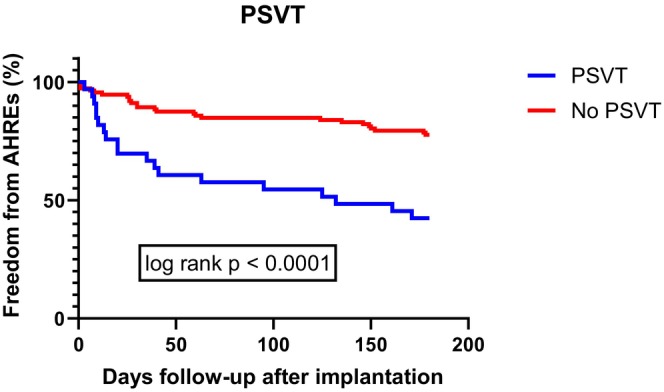
Kaplan–Meier chart of the history of paroxysmal supraventricular tachycardia and freedom from AHREs after 6‐month follow‐up.

**FIGURE 3:**
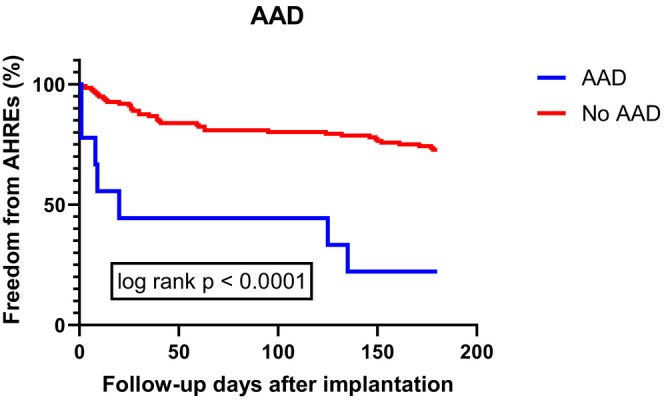
Kaplan–Meier chart of the history of anti‐arrhythmic drugs and freedom from AHREs after 6‐month follow‐up.

Univariate Cox regression analysis of the risk factors of AHRE showed that factors related to development of AHREs including using AAD before implantation [OR 5.33; CI 95% (2.36–12.05); *p* < .001], history of PSVT [OR 3.20; CI 95% (1.74–5.89); *p* < .001], the percentage of PAC on 24‐h Holter ECG monitoring before implantation [OR 1.02; CI 95% (1.01–1.05); *p* < .001] and the GLS‐LV before implantation [OR 0.44; CI 95% (0.20–0.98); *p* = .044] (Table 3; see more in Figures 2 and 3.)

**TABLE 3:** Univariate Cox regression analysis of risk factors for AHRE onset after 6‐month follow‐up.

Variables	HR	95% CI	*p*
Female	1.30	0.71–2.38	.395
Age	1.01	0.98–1.02	.861
BMI	0.97	0.88–1.07	.503
CHA_2_DS_2_‐Vasc	0.98	0.81–1.18	.817
AVB	0.60	0.31–1.16	.127
SSS	1.56	0.82–2.94	.175
ACEI/ARB	0.97	0.53–1.76	.916
MRA	0.27	0.04–1.92	.189
Statin	1.20	0.65–2.21	.561
AAD	5.33	2.36–12.05	<.001
eGFR	1.00	0.99–1.01	.972
CRP	1.00	0.99–1.01	.801
HGB	1.00	0.99–1.02	.693
WBC	1.02	0.92–1.13	.689
Ap	0.99	0.98–1.01	.366
Vp	0.99	0.98–1.00	.266
PAC (%)	1.02	1.01–1.05	.001
PSVT	3.20	1.74–5.89	<.001
E/A	1.07	0.76–1.52	.704
E/E'Ave	0.98	0.93–1.04	.582
DT	1.00	0.99–1.01	.384
LVEDD	0.99	0.950–1.05	.887
LVESD	0.99	0.94–1.04	.605
LVEF	1.02	0.99–1.06	.168
GLS_LV	0.44	0.20–0.98	.044

Abbreviations: AAD, anti‐arrhythmic drug; ACEI/ARB, angiotensin‐converting enzyme inhibitor/Angiotensin receptor blocker; Ap, atrial pacing; AVB, atrial ventricular block; BMI, body mass index; CRP, C‐reactive protein; DT, declaration time; eGFR, estimated glomerular filtration rate; GLS_LV, left ventricular global longitudinal strain; HGB, hemoglobin; LAd, left atrial diameter; LAVI, left atrial volume index; LVEDD, Left ventricular end‐diastolic dimension; LVEF, left ventricular ejection fraction; LVESD, left ventricular end‐systolic dimension; MRA, Mineralocorticoid receptor antagonists; PAC, premature atrial contraction; PSVT, paroxysmal supraventricular tachycardia; SSS, sick sinus syndrome; Vp, ventricular pacing; WBC, white blood cell.

### 3.4 Multivariate cox regression analysis of risk factors for AHRE onset after 6‐month follow‐up

Multivariable Cox regression analysis showed factors related to development of AHREs including using AAD before implantation (Hazard ratio (HR) 7.71; 95% confidence interval [95% CI], 2.58–23.02, *p* < .001), history of PSVT (HR 2.45; [95% CI], 1.18–5.09, *p* = .016), the percentage of PAC on 24‐h Holter ECG monitoring (HR 1.008; [95% CI], 1.003–1.014, *p* = .003), and the GLS‐LV (HR 0.92;[95% CI], 0.84–0.99, *p* = .049; Table 4).

**TABLE 4:** Multivariate Cox regression analysis of risk factors for AHRE onset after 6‐month follow‐up.

Variables	HR	95% CI	*p*
Age	1.00	0.98–1.02	.794
AAD	7.71	2.58–23.02	<.001
PSVT	2.45	1.18–5.09	.016
PAC (%)	1.008	1.003–1.014	.003
GLS–LV	0.92	0.84–0.99	.049

Abbreviations: AAD, anti‐arrhythmic drug; AHRE, atrial high‐rate episode; GLS‐LV, left ventricular global longitudinal strain; PAC, premature atrial contraction; PSVT, paroxysmal supraventricular tachycardia.

## 4 DISCUSSION

### 4.1 The incidence of AHRE

The incidence of AHRE in our study was 30.3% (44 cases of AHRE in 145 patients with pacemakers). This result is also consistent with 2017 consensus reports of the European Heart Rhythm Association, which is between 10 and 30%. There are several possible reasons for this^1^: atrial fibrillation is diagnosed using an ECG or Holter ECG. It is easy to miss the diagnosis when patients with paroxysmal atrial fibrillation, especially asymptomatic with low frequency and short duration.^2^ Most AHREs are asymptomatic and have short duration. They were atrial fibrillation, flutter, or atrial tachycardia. However, when other tachyarrhythmias, such as sinus tachycardia, paroxysmal supraventricular tachycardia, or ventricular tachycardia with 1:1 atrioventricular retrograde conduction, meet the diagnostic criteria for AHRE, they will be labeled as AHRE by these devices.^3^ A false‐positive diagnosis of AHRE can also result from atrial over‐sensing, R wave far‐field phenomena, or other external signal disturbances.

The results of our study are similar to the research of author Hoang Quynh Hue and colleagues published in 2021.^15^ The incidence of AHRE in Hoang Quynh Hue's study was 32.8% higher than ours, but not much. This is possibly because the study authors followed up for a longer period than us (1 year). Our study followed all the patients for 6 months after implantation.

### 4.2 Anti‐arrhythmic drugs

In the Hideyuki Kishima study published in 2020, the author found that ß‐blockers were a risk factor for AHRE in univariate analysis with HR 2.12; 95% confidence interval (1.10–4.08), *p* = .035.^16^ Another valuable study is the subgroup analysis of the MOST study^17^ with 312 patients. The incidence of using anti‐arrhythmic drugs at hospital admission was 29% in the AHRE group compared with only 11% in the group without AHRE, *p* = .001 < .05; the difference was statistically significant. Our study's results are similar to the findings of Kishima and the subgroup MOST study.^17^ This could be explained as patients with a history of taking anti‐arrhythmic drugs for the treatment of prior atrial arrhythmias having more structural or electrical abnormalities. Patients will easily develop AHRE, which may be atrial tachycardia, atrial flutter, subclinical atrial fibrillation, etc.

### 4.3 The percentage of pre‐implantation PACon 24‐h Holter ECG monitoring

In a multicenter study on the frequency and risk factors of AHRE on 816 patients with dual‐chamber pacemakers by Kim Min published in 2020, he found that the rate of PAC above 1% detected on Holter ECG before implantation was a risk factor for AHRE. The rate in the AHRE group was higher than the No AHRE group (14.3% vs. 5%), *p* = .005.^18^ The findings in our study are similar to those of author Kim Min. We know that the definition of AHRE is any atrial tachycardia that is >190 bpm. The greater the incidence of atrial ectopic beats in patients, the more likely they develop AHRE. This reflects the greater substrate damage and scarring in the atrium.

### 4.4 The history of PSVT

In a subgroup analysis of the MOST study including 312 patients, of whom 160 patients had AHRE and 152 did not, the rate of pre‐implantation PSVT was 81% in the AHRE group compared to only 39% in the group without AHRE, *p* = .001; differences are statistically significant. Our findings are similar to those drawn from the MOST subgroup study published in the journal *Circulation*. This is one of the studies that paved the way for understanding AHRE to this day.

### 4.5 Left ventricular global longitudinal strain

GLS‐LV predicts atrial fibrillation risk even in the absence of structural heart disease and other clinical risk factors. Our ambition is to take it one step further. Using GLS‐LV to predict an early form of atrial fibrillation is subclinical atrial fibrillation. To our knowledge, this may be the first study to report the relationship between GLS‐LV and AHRE in Vietnam and possibly worldwide.

A cohort study by Russo et al. showed that GLS‐LV is a notable predictor of atrial fibrillation in the elderly population.^19^ In the atrial fibrillation group, the GLS‐LV was −17.2 ± 3.0 compared with the group without atrial fibrillation of −15.2 ± 4.1; *p* < .001 is very statistically significant. The author also analyzed the subgroup analysis with the GLS‐LV cutoff index of −14.7%. The GLS‐LV ≥ −14.7% accounted for 16.8% in the group without atrial fibrillation compared with 46.9% in the atrial fibrillation group, *p* < .001.^19^ The study by author Flemming Javier Olsen was published in August 2021 on 1309 patients. The results showed that abnormal GLS‐LV increased the risk of atrial fibrillation more than two times (HR = 2.16; 95% CI (1.26–3.72)) compared with GLS‐LV within normal limits.^20^ However, the author also reported that only about 60% of patients in the study could measure GLS‐LV; many patients could not be measured due to older age, high BMI, and severe underlying cardiovascular disease.^20^ Compared with our study, the proportion of patients who measured GLS‐LV was 122/145, reaching 85%. This shows our great effort when comparing two population samples that are not very different and proves that this study is highly valuable.

To our knowledge, this is the first study to report an association between GLS‐LV and AHRE or subclinical atrial fibrillation. Most of the current literature reports an association between GLS‐LV and clinical atrial fibrillation. This makes this study even more valuable. Our study found that GLS‐LV is the most remarkable echocardiographic marker to predict AHRE in patients with dual‐chamber pacemaker implantation. Therefore, we believe GLS‐LV is a simple and useful predictor of AHRE in normal structural hearts undergoing implantation. Our findings may provide additional prognostic information for other factors. However, the management of patients with AHRE detected by intracardiac ECG remains controversial. Current guidelines do not specifically address the management of these patients. Further studies are ongoing to confirm the benefit of oral anticoagulants in patients with AHRE. And that will be our next research direction in the future.

## 5 CONCLUSIONS

The incidence of AHRE after 6‐month follow‐up was 30.3%. In which history of AAD, history of PSVT, percentage of PAC on 24‐h Holter ECG monitoring before implantation, and GLS‐LV are the independent predictors for AHRE.

## 6 LIMITATIONS

The current study has several limitations. This was a cohort study with a small number of patients and a short follow‐up period. Therefore, it may not reflect all independent predictors of AHRE occurrence, and may be influenced by other confounding risk factors, which are not currently collected. The generalizability of these results is also limited because data were collected from only a single center. Although every effort has been made to accurately determine AHRE using intracardiac electrogram data, it is not possible to 100% rule out the possibility of errors due to oversensing or far‐field R wave phenomena. We found nine patients using AAD in the main analysis, but the number is small and we confirmed that they were not used for heart failure or hypertension purposes. Therefore, it is judged that the impact of this part on the main outcome is minimal.

## CONFLICT OF INTEREST STATEMENT

None of the authors have any conflicts of interest to declare.

## ETHICS STATEMENT

This study was conducted in accordance with the ethical standards laid down in the 1964 Declaration of Helsinki and its later amendments. This study has been approved by the ethics committee on biomedical research at Pham Ngoc Thach University of Medicine according to Official Letter No. 445/HDĐ‐ĐHYKPNT dated December 11, 2020.

## PATIENT CONSENT STATEMENT

All patients provided written informed consent after receiving full details of the study, including its purpose, procedures, and potential risks. Participation was voluntary, with the option to withdraw at any time.

## Data Availability

All data used in this study are not publicly available according to Cho Ray hospital's policies. Any queries surrounding this study's data can be sent to the corresponding author's email.
